# Fatal necrotizing pneumonia due to a Panton-Valentine leukocidin positive community-associated methicillin-sensitive *Staphylococcus aureus *and Influenza co-infection: a case report

**DOI:** 10.1186/1476-0711-7-5

**Published:** 2008-02-19

**Authors:** Jill C Roberts, Sam P Gulino, K Kealy Peak, Vicki A Luna, Roger Sanderson

**Affiliations:** 1 Center for Biological Defense, College of Public Health, University of South Florida, Tampa Florida, USA; 2 College of Medicine, University of South Florida and Hillsborough County Medical Examiner Department, Tampa Florida, USA; 3 Bureau of Laboratories – Tampa, Florida Department of Health, Tampa, Florida, USA

## Abstract

Recent studies have described a number of fatalities due to methicillin-resistant *Staphylococcus aureus *(MRSA) and influenza virus co-infection. MRSA isolates provide a challenge to caregivers due to inherent wide range antibiotic resistance. Many facilities have instituted screening methods, based on the presence of antibiotic resistance genes, to identify MRSA positive patients upon admission. However, the resistance profile of the pathogen does not necessarily determine the severity of disease caused by that organism.

We describe a fatal case of necrotizing pneumonia in a patient co-infected with Influenza B and a community-associated, PVL-positive methicillin-susceptible *Staphylococcus aureus *(MSSA).

## Background

Necrotizing pneumonia due to community-associated methicillin-resistant *Staphylococcus aureus *(CA-MRSA) is increasingly reported in otherwise healthy individuals [1]. Several cases of community-associated pneumonia (CAP) have been attributed to methicillin-resistant strains such as the USA 300 epidemic CA-MRSA clone and are often associated with influenza virus infection or influenza-like illness (ILI) [2]. The USA 300 clone harbors the gene encoding Panton-Valentine leukocidin (PVL) [3], a pore-forming toxin that targets cells of the immune system [4]. Although the majority of CA-MRSA infections involving skin and soft tissue likely occur independent of PVL toxin production [5], PVL is required for lung tissue necrosis and inflammation as present in the rare, but frequently fatal, cases of necrotizing pneumonia[4,6]. We report here a fatal case of necrotizing pneumonia in a patient co-infected with Influenza B and a PVL-positive *Staphylococcus aureus *that in contrast to recent reported cases is methicillin-susceptible.

## Case presentation

A previously healthy 30 year old black woman went to her local emergency department on April 12, 2007 with a 4 to 5 day history of sore throat, chills, fever and shortness of breath. A chest radiograph was performed and read as clear, however detail was obscured by abundant overlying soft tissue. She was diagnosed with bronchitis and treated with intravenous antibiotics and steroids. She was discharged to home with a prescription for albuterol and azithromycin, however the antibiotic was not utilized. Early the following morning, she developed worsening symptoms including hemoptysis. She was brought to the emergency room by ambulance. Vital signs at time of admission included a fever of 100.5°F (38°C), pulse of 165 bpm, and blood pressure of 104/53 mm Hg. In the emergency room, she developed hypotension and respiratory failure requiring intubation. A chest x-ray revealed bilateral pulmonary infiltrates and a right pleural effusion. She had a pronounced leukopenia (0.9/mm^3^) with 53% neutrophils. She was admitted to the intensive care unit with a diagnosis of septic shock with hemorrhagic pneumonia, and was treated with ceftriaxone and moxifloxacin. Additional antibiotics included gentamycin, ciprofloxacin, piperacillin, and tazobactam. The platelet count was initially normal but dropped quickly after admission. Despite aggressive care, including vasopressors and additional antibiotics, she died that night. A blood culture collected prior to death was positive for *Staphylococcus aureus*. The culture was resistant to amoxicillin, clarithromycin, erythromycin, and penicillin, and susceptible to the rest of the panel including oxacillin. The Florida Department of Health was notified of the death, as was the Hillsborough County Medical Examiner. An autopsy was performed the following day which revealed bilateral necrotizing bronchopneumonia with abscess formation and diffuse alveolar damage. Clusters of cocci were seen in alveoli along with macrophages. Focally, there was necrosis of blood vessel walls, associated with intra-alveolar hemorrhage. Larger bronchi revealed acute mucosal inflammation and epithelial necrosis. Acute tubular necrosis of the kidneys was also noted.

## Methods and results

**Figure 1:**
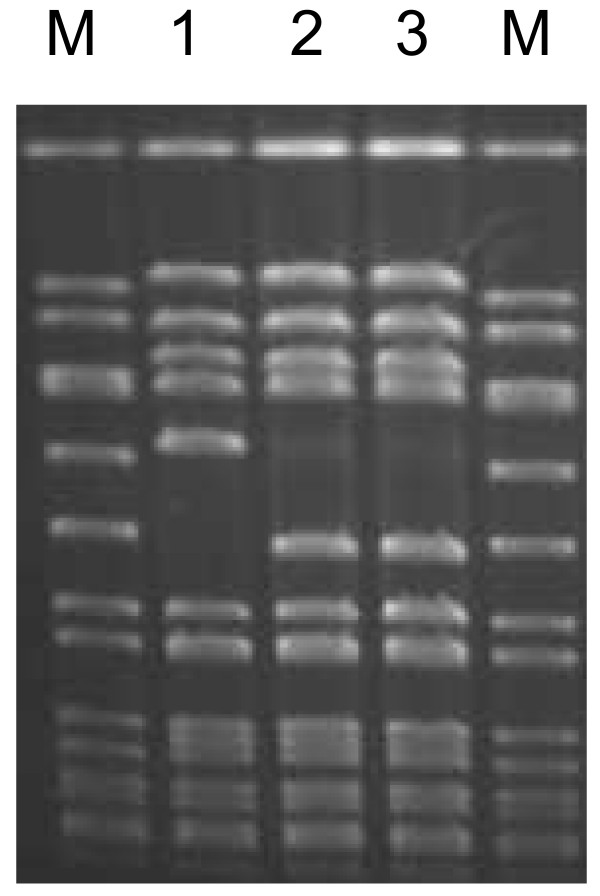
**Pulsed-field gel electrophoresis.** Lanes are as follows; lane M: *S. aureus *standard *Sma*I digested strain 8325, Lane 1: USA300 control strain, Lanes 2 and 3, patient blood and lung samples, respectively.

Specimens from blood, right and left lung were sent to the Florida Department of Health laboratory in Tampa. Viral studies were positive for influenza B using an RT-PCR protocol provided by the Centers for Disease Control and Prevention (Lindstrom, personal communication). Assay specifications are available from CDC upon request. *S. aureus *was cultured from blood and in tissue from both lungs. Molecular genotyping of the *S. aureus *isolate was performed using pulsed-field gel electrophoresis (PFGE) and multi-locus variable number tandem repeat fingerprinting (MLVF) as previously described [7,8]. The resulting pulsotypes, which were identical for the three patient samples, were compared to USA 300 (Figure 1) and over 400 *S. aureus *isolates in our database (data not shown) and no match was identified. Comparison of the MLVF pattern with common CA-MRSA patterns also demonstrated no known match. The disease progression suggested that this patient died of co-infection with CA-MRSA and influenza. However, the lack of a matching PFGE pattern in our database which consists of more than 400 CA-MRSA isolates collected in Florida over the past 5 years [7] led us to suspect this isolate was not methicillin-resistant. Disk diffusion susceptibility testing was performed as previously described [9] and the isolate proved to be susceptible to both oxacillin and cefoxitin. Penicillin-binding protein 2' detection was performed using a slide agglutination test (Denka Seiken Co, LTD., Tokyo, Japan), and PCR for the *mecA *gene was performed as previously described [10]. Results from these three tests demonstrated that the patient's isolate lacks both the protein product and the *mecA *gene. Microbroth dilution and Etest also confirmed the isolate is susceptible to oxacillin confirming its identity as a methicillin-susceptible *Staphylococcus aureus *(MSSA). PCR performed as previously described [11] demonstrated that the isolate was positive for the PVL toxin genes.

## Conclusion

This case was the fourth case of fatal community associated *S. aureus *pneumonia co-infected with influenza B reported to the Florida Department of Health from January 1, 2007 through April 30, 2007. All four cases were reported from Hillsborough County Florida, 2007 estimated population 1,188,706. In contrast to the case presented herein, the three previous cases were confirmed MRSA and each was USA300 by PFGE. These cases age 4, 14 and 18 had similar presentations and clinical course to the MSSA case described here.

Fatal cases of community-acquired pneumonia due to co-infection with influenza and MRSA have increasingly been reported among previously healthy individuals [1]. In a recent morbidity and mortality weekly report, Jones-Nazar et al. [1] reported 10 cases of MRSA CAP with preceding or concurrent ILI in Louisiana and Georgia in which 60% of the patients succumbed to the illness. Severe CAP was also reported in nine states during the 2003–04 influenza season, 71% of cases with demonstrated influenza laboratory confirmation [2]. Previous studies have reported fatal cases of necrotizing pneumonia due to MSSA in patients with a history of influenza-like syndrome [12]. Studies of pneumonia caused by PVL-positive MRSA and MSSA have demonstrated a number of factors which predict mortality [12,13]. These factors included airway bleeding and leukopenia [12,13], both of which were reported clinical findings in the case described herein. In addition, the patient required mechanical ventilation following respiratory failure which has also been associated with fatal outcomes [13].

To our knowledge, our patient represents the first fatal case of community-acquired pneumonia in which co-incident infection with both influenza B and PVL-positive MSSA was laboratory confirmed.

Although MRSA is an uncommon cause of CAP, the Centers for Disease Control and Prevention has initiated active population-based surveillance for invasive MRSA disease in several locations in the United States [2]. A number of healthcare facilities have initiated methods to screen incoming patients for MRSA using nasal swabs and commercially available media such as MRSAID (bioMerieux), MRSA *Select *(Bio-Rad Laboratories), and CHROMagar MRSA (Becton Dickinson Microbiology) [14]. Detection of MRSA from nasal swabs can also be performed using molecular techniques such as IDI-MRSA (GeneOhm Sciences, Sheffield, UK), and other PCR-based techniques. However, selective media are inhibitory to MSSA and the organism does not possess the SCC *mec *target used for many of the PCR reactions. Therefore, colonization and/or infection with MSSA may be missed upon screening.

This case report illustrates that *S. aureus *isolates containing highly virulent toxin genes, such as PVL, may be deadly in patients co-infected with influenza, regardless of the *S. aureus *antibiotic susceptibilities.

## Competing interests

The author(s) declare that they have no competing interests.

## Authors' contributions

JCR performed PFGE, MLVA, PVL PCR, and authored manuscript. SPG provided autopsy information and authored autopsy section of manuscript. KKP performed disk diffusion, PBP2a testing. VAL performed microbroth dilution. RS provided chart review and authored case history and country epidemiology section of manuscript. All authors read and approved the final manuscript.

## Consent

This case report represents a fatal case of disease. The data presented herein was provided with all clinical identifiers removed as per HIPAA guidelines. As such, the identification of the patient is unknown to the authors.
